# Regulation of Posttranscriptional Modification as a Possible Therapeutic Approach for Retinal Neuroprotection

**DOI:** 10.1155/2011/506137

**Published:** 2010-11-07

**Authors:** Yoko Ozawa, Toshihide Kurihara, Kazuo Tsubota, Hideyuki Okano

**Affiliations:** ^1^Laboratory of Retinal Cell Biology, Keio University School of Medicine, 35 Shinanomachi, Shinjuku-ku, Tokyo 160-8582, Japan; ^2^Department of Ophthalmology, Keio University School of Medicine, 35 Shinanomachi, Shinjuku-ku, Tokyo 160-8582, Japan; ^3^Department of Physiology, Keio University School of Medicine, 35 Shinanomachi, Shinjuku-ku, Tokyo 160-8582, Japan; ^4^Solution Oriented Research for Science and Technology (SORST), Japan Science and Technology Corporation (JST), 4-1-8 Honcho, Kawaguchi, Saitaima 332-0012, Japan

## Abstract

Understanding pathogenesis at the molecular level is the first step toward developing new therapeutic approaches. Here, we review the molecular mechanisms of visual dysfunction in two common diseases, innate chorioretinal inflammation and diabetic retinopathy, and the role of the ubiquitin-proteasome system (UPS) in both processes. In innate chorioretinal inflammation, interleukin-6 family ligands induce STAT3 activation in photoreceptors, which causes UPS-mediated excessive degradation of the visual substance, rhodopsin. In diabetic retinopathy, angiotensin II type 1 receptor (AT1R) signaling activates ERK in the inner layers of the retina, causing UPS-mediated excessive degradation of the synaptic vesicle protein, synaptophysin. This latter effect may decrease synaptic activity, in turn adversely affecting neuronal survival. Both mechanisms involve increased UPS activity and the subsequent excessive degradation of a protein required for visual function. Finally, we review the therapeutic potential of regulating the UPS to protect tissue function, citing examples from clinical applications in other medical fields.

## 1. Introduction

Recent progress in molecular biology has revealed the molecular basis in the pathogenesis of various diseases. Molecular targeting therapies have been developed, primarily in the field of vascular biology. One such therapy is antivascular endothelial growth factor (anti-VEGF) therapy, which is now widely used to treat age-related macular degeneration (AMD) and cancer. Its role in treating AMD is to regulate ocular vascular lesions and prevent secondary damage to the neural retinal cells, which are critical for visual function. 

The first research into VEGF was reported in the 1970s [1], and in 2004 the FDA approved the first anti-VEGF drug for clinical use in human eyes [2]. Basic research on neurotrophic regulation also began in the 1970s [3], but clinical trials started only recently [4]. Molecular-targeting therapies for retinal neuroprotection are on the horizon, and further studies are needed to understand the molecular mechanisms in retinal diseases and to explore new treatment approaches.

In the treatment of retinal diseases, developing neuroprotective therapies for neural retinal cells deserves special emphasis; these cells have a very limited regenerative capacity and are critical to vision. The neural retinal cells derive from the monolayer of the neural tube during embryogenesis and are part of the central nervous system. Damage to these cells occurs in common diseases such as chorioretinal inflammation and diabetic retinopathy, as well as in less-common conditions, like retinitis pigmentosa, a hereditary retinal degeneration with mutated genes in the retinal cells. Severe chorioretinal inflammation acutely disturbs visual function [5]. Diabetes chronically affects it, even in the absence of obvious microangiopathy [6–8]: patients experience a gradual loss of visual function even when diabetic neovascularization is well regulated by vitreous surgery and/or anti-VEGF therapy. In AMD, local retinal inflammation is involved in the process of vision loss; association of inflammatory molecules is reported in both early and late stage AMD [9]. Inflammatory cytokines can play a role in most of these changes. 

However, the investigation of the molecular mechanisms of retinal neuropathogenesis is in its early stages. Here, we describe the molecular mechanism of neurodegeneration that we recently reported in animal models of innate chorioretinal inflammation (endotoxin-induced uveitis) and diabetic retinopathy, and compare our findings with studies from other fields to obtain additional clues to the pathogenesis of retinal diseases.

## 2. Retinal Neuronal Changes in Innate Chorioretinal Inflammation

Inflammatory cytokines such as interleukin-6 (IL-6) are closely connected to retinal diseases. Clinical reports show that IL-6 in the vitreous fluid increases not only in uveitis [10] but also in diabetic retinopathy [11, 12], retinal vein occlusion [13], and retinal detachment [14]. 

### 2.1. IL-6 Family Ligands and STAT3/SOCS3 Pathway in the Retina

Research with experimental animals has shown that diffusible factors, IL-6 and other proteins in the IL-6 family, such as leukemia inhibitory factor (LIF) and ciliary neurotrophic factor (CNTF), are expressed in the retina. Both IL-6 [15] and LIF [16] are found in Müller glial cells, and CNTF is found in the retinal ganglion cells and astrocytes around the vessels [17]. These endogenous IL-6 family proteins are upregulated during inflammation and function to promote pathogenesis of the vascular system [18].

**Figure 1:**
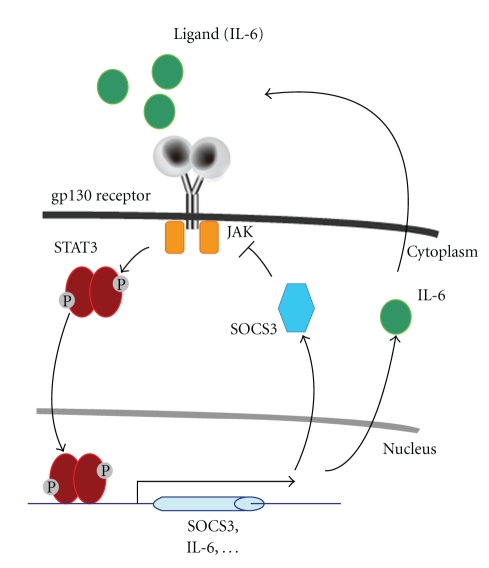
*Model of the gp130-STAT3-SOCS3 pathway.* IL-6 family ligands activate the gp130 receptor, which subsequently phosphorylates and activates STAT3 through JAK. Activated and dimerized STAT3 translocates into the nucleus to promote the transcription of various molecules, including SOCS3 and IL-6. SOCS3 inhibits JAK and STAT3 activation. IL-6 is secreted and further activates STAT3.

IL-6 family proteins use cytokine-specific receptors to activate a transmembrane receptor, gp130 [19], which then recruits Janus kinase (JAK) to activate transcription factor signal transducer and activator of transcription 3 (STAT3). STAT3 then regulates various molecules at the transcriptional level, including suppressor of cytokine signaling 3 (SOCS3). SOCS3 acts as a negative feedback modulator of STAT3 by inhibiting JAK and subsequent STAT3 activation [20] (Figure 1). In the retina, SOCS3 is expressed in the photoreceptor cells, Müller glial cells, and retinal ganglion cells, and it inhibits STAT3 activation in these cells [21, 22]. Since STAT3 activation induces further STAT3-activating factors, such as the IL-6 family ligands [23], the balance between STAT3 activation and SOCS3 level is one of the key determinants of an inflammatory reaction [23, 24].

### 2.2. STAT3/SOCS3 Pathway in the Developing Retina

This balance between STAT3 and SOCS3 also plays an important role during the development of the retina; activated STAT3 inhibits the photoreceptor-specific transcription factor crx at the transcriptional level, which in turn inhibits downstream photoreceptor-specific markers such as rhodopsin [25]. Retina-specific conditional knockout mice of *SOCS3*, *α*-Cre SOCS3 flox/flox mice (SOCS3CKO), induce increase in the endogenous STAT3 activation, and show delay in the initiation of rhodopsin expression at the transcriptional level [22]. STAT3 is activated in the embryonic retina but is shut down by SOCS3 which appears in the neonatal retina, thereby allowing rod photoreceptor cell differentiation. Therefore, the timing of rod photoreceptor cell differentiation is fine-tuned by the initiation of SOCS3 expression and downregulation of STAT3 activation. Although STAT3 activation in the SOCS3CKO retina is still upregulated in the adulthood, the rhodopsin level in the SOCS3CKO mice is compensated for by as-yet-unknown mechanisms and matches that of wild-type mice.

### 2.3. STAT3/SOCS3 Pathway in the Adult Retina with Inflammation

A murine model of accelerated innate immunity, the endotoxin-induced uveitis model (EIU model), was used to further analyze the role of the gp130-STAT3-SOCS3 loop in adult retinal inflammation. In this model, uveitis is induced by administering lipopolysaccharide (LPS). Inflammatory cytokine, IL-6 is upregulated [18], leading to STAT3 activation [21, 26, 27] in the retina. This induction does not cause retinal cellular apoptosis, but it does reduce visual function [21, 26, 27]: rhodopsin protein levels decrease, and the rod photoreceptor outer segments (OSs), where rhodopsin is concentrated, are shortened. The scotopic electroretinogram (ERG) a-wave amplitude, which represents rod photoreceptor cell function, also decreases. STAT3 activation correlates with the rhodopsin reduction in the adult retina, as in the developing retina. Surprisingly, though, neither rhodopsin mRNA nor its upstream regulator, crx, decreases in the adult retina during inflammation. This suggests that a different mechanism is involved in rhodopsin and crx regulation in the adult retina than in the developing retina [22, 25]. 

**Figure 2:**
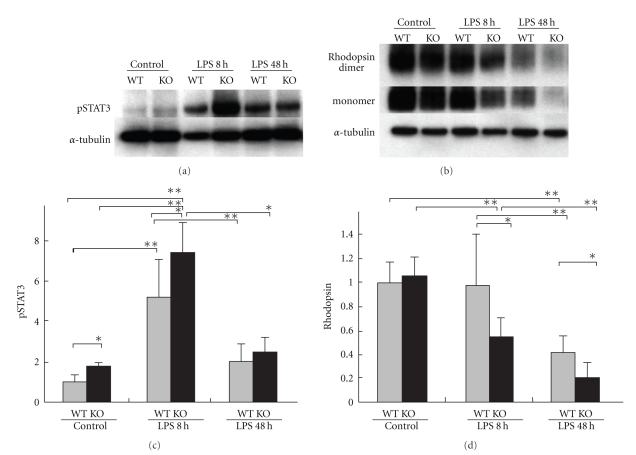
*STAT3 activation and rhodopsin protein levels in the retina of EIU model generated in SOCS3CKO mice.* Immunoblot analyses. In the SOCS3CKO retina, phosphorylated and activated STAT3 are upregulated in the control condition, and more greatly increased in the EIU model induced by LPS (a, c). Although the rhodopsin protein level in the adult SOCS3CKO retina is almost the same as wild-type retina under control condition, its level was more severely reduced in the EIU model induced by LPS (b, d). WT: wild-type; KO, SOCS3CKO; LPS: lipopolysaccharide; pSTAT3: phosphorylated STAT3; EIU: endotoxin-induced uveitis. This research was originally published in *J Biol Chem*. Ozawa Y, et al. Roles of STAT3/SOCS3 Pathway in Regulating the Visual Function and Ubiquitin-Proteasome-dependent Degradation of Rhodopsin during Retinal Inflammation. *J Biol Chem*. 2008; 283(36):24561–24570. the American Society for Biochemistry and Molecular Biology.

The role of activated STAT3 in retinal dysfunction during inflammation has been analyzed using SOCS3CKO mice [21]. In these *SOCS3*-deficient mice, STAT3 activation can increase greatly in the retina (Figures 2(a) and 2(c)). Thus, we have hypothesized that the mechanism of rhodopsin reduction during inflammation might be enhanced in these cells. 

As expected, the EIU models generated in the adult SOCS3CKO mice showed a relative depletion of rhodopsin protein (Figures 2(b) and 2(d)), followed by OS shortening. The subsequent rod photoreceptor cell dysfunction, as measured by scotopic ERG, was prolonged. This model also revealed that during inflammation, rhodopsin reduction is not regulated at the transcriptional level, but by a post-transcriptional inhibitory mechanism. The reduction in rhodopsin protein levels is rapid and global, starting only several hours from the onset of inflammation.

### 2.4. STAT3-Induced Rhodopsin Degradation through UPS

Under stress conditions, massive protein degradation through the ubiquitin-proteasome system (UPS) is known to increase [28]. A genetically abnormal rhodopsin protein that causes autosomal dominant retinitis pigmentosa, P23H, interacts with the UPS and forms aggresomes when overexpressed in a cell line [29, 30]. Aggresomes are inclusion bodies of accumulated waste proteins, formed when cellular degradation machinery is impaired or overwhelmed, and they are a pathologic finding in neurodegenerative diseases. In the case of P23H, the rhodopsin protein folds abnormally and accumulates rather than following the normal process of elimination from the cell. This finding hinted that genetically normal rhodopsin protein might also be degraded extensively following the excessive induction of the UPS by inflammation. Moreover, ubiquitin is present in the rod OS under control conditions [31], thus it can rapidly degrade rhodopsin as needed. This hypothesis has been clearly verified *in vivo* and *in vitro* [21]. Elevated levels of ubiquitin-conjugated rhodopsin are followed by rhodopsin depletion in the SOCS3CKO EIU mouse model. The same process occurs in wild-type mice, but it is more rapid and more severe in the SOCS3CKO mice, in which STAT3 activation is increased. Therefore, the activated STAT3 level correlates with the ubiquitination and degradation of rhodopsin. 

**Figure 3:**
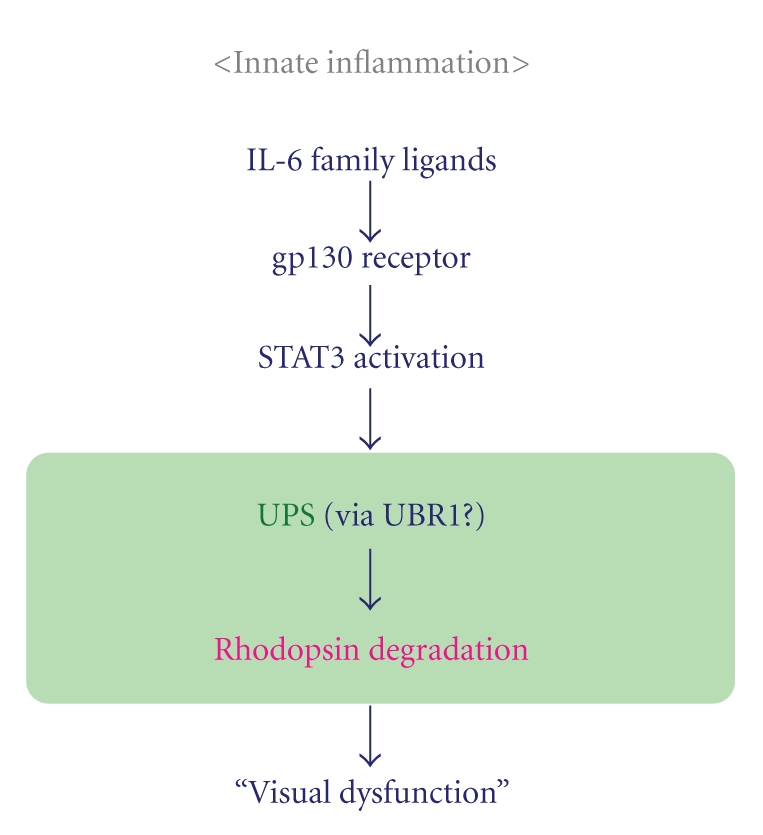
*Model of the molecular mechanism in retinal inflammation.* IL-6 family ligands induce STAT3 activation, which promotes excessive UPS-mediated rhodopsin protein degradation and subsequent visual dysfunction.

This has been confirmed *in vitro* by using JAK inhibitor to inhibit IL-6-induced STAT3 activation. It is illustrated by the preservation of rhodopsin levels under IL-6-induced STAT3 activation, when a STAT3-dependent ubiquitin E3 ligase, ubiquitin-protein ligase E3 component n-recognin 1 (UBR1), is inhibited through the small inhibitory RNA (siRNA) system. UBR1 is expressed in the OSs. We propose that it contributes to rhodopsin protein degradation during inflammation, especially given that inflammatory cytokines, including IL-6, LIF, and CNTF, induce the ubiquitin-conjugation of rhodopsin protein and UBR1 expression in the rod photoreceptor cells, resulting in excessive rhodopsin degradation and disturbed visual functioning (Figure 3).

### 2.5. Other Molecules Related to STAT3 Activation during Inflammation

This process lasts as long as STAT3 is activated. STAT3 can be activated not only through the gp130 receptor, but also through an inflammatory diffusible factor, angiotensin II. An angiotensin II type 1 receptor blocker (ARB) suppresses STAT3 activation [26] during inflammation directly, or indirectly, inhibiting IL-6 production, thereby preserving rhodopsin levels and visual function. Angiotensin II also induces oxidative stress, which can induce the ubiquitination of specific proteins [32]. The antioxidant lutein, which suppresses oxidative stress and the induction of reactive oxygen species (ROS) during inflammation, also reduces STAT3 activation, thus preserving the rhodopsin level and visual function during inflammation [27]. 

Therefore, the mechanism of visual dysfunction is, at least in part, explained by the excessive degradation of the essential protein during inflammation.

## 3. Retinal Neuronal Changes in Diabetes

**Figure 4:**
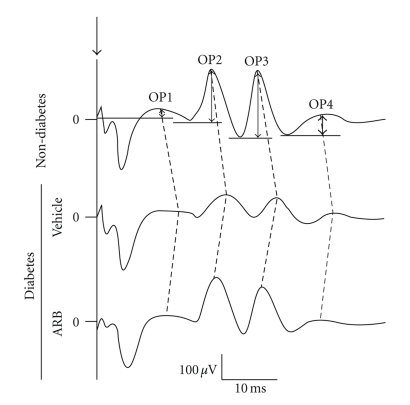
*Impairment of visual function and the protective effect of ARB in diabetic mice.* OPs in ERG from diabetic or nondiabetic mice. Amplitude and implicit time of OPs are impaired in the diabetic mice, but these changes were avoided by administrating ARB (Telmisartan). ERG: electroretinogram; Ops: oscillatory potentials; ARB: Angiotensin II type 1 receptor blocker.

In diabetic retinopathy, the main findings include microangiopathy [33, 34] and neurodegeneration [35, 36]. Visual dysfunction begins before vascular abnormalities become obvious [6–8], and inner retinal dysfunction is reflected in changes in ERG oscillatory potentials (OPs) (Figure 4). However, little is known about the molecular mechanism of diabetes-related neuronal degeneration. Our recent analyses using a streptozotocin- (STZ-) induced type 1 diabetes model shed light on this critical issue [35, 36].

### 3.1. Angiotensin II and Its Type1 Receptor Signaling

Both diabetes and hypertension are involved in metabolic syndrome, in which angiotensin II signaling plays an important role. ARBs have been approved for treatment of not only high blood pressure, but also diabetes-related renal failure [37]. Angiotensin II is converted from angiotensinogen in a stepwise fashion by enzymes, including renin, angiotensin converting enzyme, and others. Angiotensin II can bind to either the angiotensin II type 1 receptor (AT1R) or type 2 receptor (AT2R) on the cell surface, which in turn activates several contextually-dependent intracellular signals. These components of the rennin angiotensin system (RAS) are all present physiologically in the retina, and are upregulated in pathological conditions, as we have shown in the murine model retina of STZ-induced diabetes [35].

AT1R is coexpressed with the major synaptic vesicle protein synaptophysin in the inner layers of the retina [26]. This is consistent with several previous reports showing the synaptic expression of AT1R in the brain [38, 39]. Synaptophysin, a synapse marker, is reduced in the postmortem brains of patients who had had neurodegenerative diseases such as Parkinson's disease and Alzheimer's disease [40]. Given that the OPs in ERG originate from inner retinal neurons bearing AT1R, these ERG changes may represent angiotensin II-induced synaptophysin dysregulation and the resulting damage to visual function. 

We have verified this hypothesis by administering ARB (either telmisartan or valsartan) to STZ-induced diabetic mice [35]. ARBs protect the expression of synaptophysin protein and OPs in the diabetic retina (Figure 4). Interestingly, synaptophysin mRNA is not reduced in the diabetic retina, indicating that the protein's reduction is regulated post-transcriptionally.

### 3.2. AT1R-Mediated Synaptophysin Degradation through UPS

Post-transcriptional synaptophysin reduction caused by angiotensin II exposure was reproduced in a rat neuronal cell line, PC12 [35]. In this system, synaptophysin protein degradation is inhibited by the proteasome inhibitor MG132, but not the lysosome inhibitor E64. AT1R and its downstream extracellular signal-related protein kinase (ERK) activation induce synaptophysin degradation, and AT1R increases the ubiquitin-conjugated synaptophysin protein levels. Angiotensin II signaling activates ERK in the diabetic retina *in vivo*, suggesting that the AT1R-ERK pathway is responsible for diabetes-induced pathogenic protein degradation through the UPS. 

Synaptophysin protein may be degraded by the mammalian homolog of *Drosophila* seven in absentia (sina), an E3-ligase selective for synaptophysin named seven in absentia homologue (Siah). Since *Drosophila *sina is regulated by ERK signaling [41], the Siah may also be regulated by ERK activation, which is increased in the diabetic retina. ERK activation and the resulting reduction of synaptophysin in the diabetic retina is also inhibited by an antioxidant, lutein [36], which indicates that angiotensin II signaling and oxidative stress may share a role in the pathogenesis of diabetic retinopathy.

### 3.3. Influence of Synaptophysin Depletion

**Figure 5:**
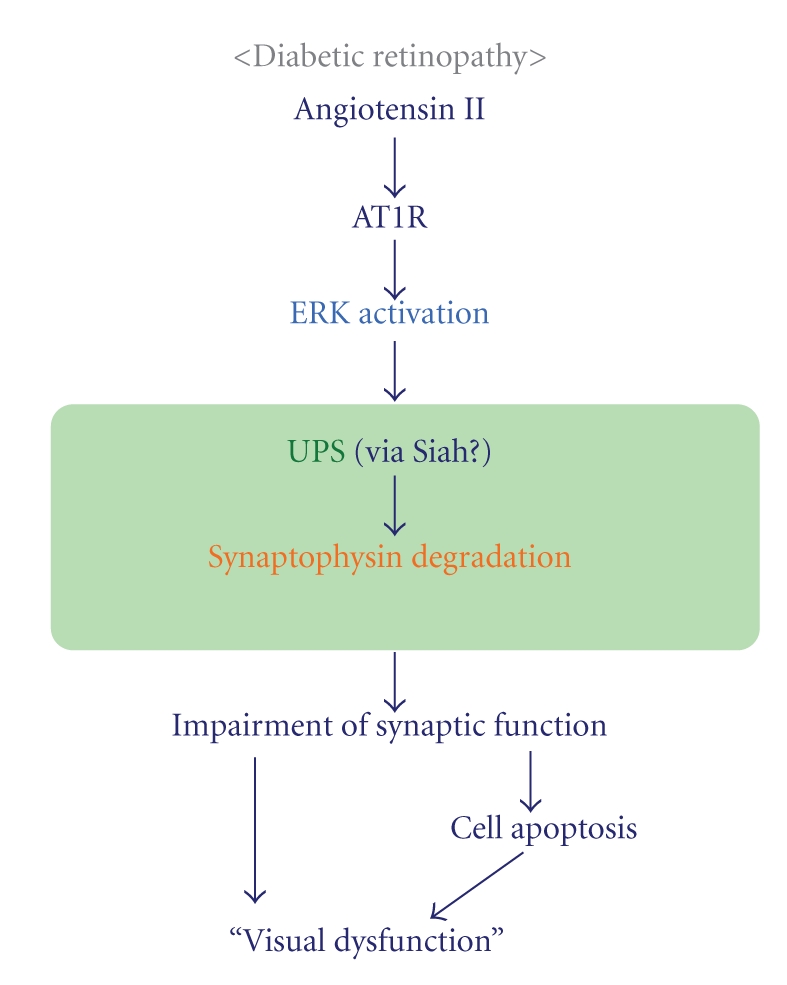
*Model of the molecular mechanism in diabetic retinopathy.* Angiotensin II binds to AT1R and induces ERK activation, resulting in excessive UPS-mediated synaptophysin protein degradation. This impairs synaptic activity, which is critical for visual function and neuronal cell survival.

Not only that the reduction of synaptophysin, a synaptic protein, impairs the transmission of neuronal and visual signals, but impairment of synaptic activity itself inhibits neuronal cell survival [34, 35]. Synaptic activity, that is, the neuronal electric stimuli, directly increases the levels of intracellular calcium ion in neurons, which promotes cell survival. Moreover, brain-derived neurotrophic factor, BDNF, a neuronal survival factor, is regulated by neuronal synaptic activity [37, 38]. Taken together, these findings suggest that the synaptophysin levels and the related neuronal synaptic activity function together to influence neuronal survival and neuronal network activity. The reduction of synaptophysin levels and neuronal activity, observed 1 month after the onset of diabetic retinopathy, is later followed by the apoptosis of retinal ganglion cells and inner retinal cells [36]. Therefore, one part of the neurodegenerative mechanism in the diabetic retina is explained by the excessive degradation of a protein that is essential for visual function (Figure 5). 

## 4. Dysregulation of UPS in Pathogenesis

The UPS is a rapid and effective method of degrading specific proteins, and in many cases a protein is degraded only in response to a particular cellular signal or event [42]. Ubiquitin molecules are attached to targeted proteins and variably elongated. This process involves the coordinated actions of three enzymes—a generally distributed E1 ubiquitin-activating enzyme, several more specific E2 ubiquitin-conjugating enzymes, and highly specific E3 ubiquitin ligases for the targeted protein. 

### 4.1. Excessive Degradation of Proteins Essential for Tissue Function

The UPS involvement in pathogenesis has led to interest in targeting proteasomes as a therapeutic approach in several fields. UPS is involved in cardiomyocyte cell pathogenesis: oxidized and ubiquitinated proteins are observed in rat hearts after cardiac ischemia/reperfusion injury [43, 44]. This may indicate the excessive degradation of proteins that are needed in muscle contraction. Muscle wasting due to UPS activation is also reported in cases of chronic kidney disease, diabetes, high angiotensin II levels, and sepsis, all of which cause inflammation, inhibit insulin signaling, and promote glucocorticoid expression to induce protein degradation [45–47]. This pathway can be blocked by overexpressing IGF-1, which inhibits atrogin-1, an E3-ligase acting for muscle atrophy, through PI3K/AKT [46, 48]. 

In the retina, innate inflammation activates the IL-6-STAT3 pathway, and diabetes activates the angiotensin II-ERK pathway. Both pathways induce the UPS, most likely through inducing a specific E3-ligase. Moreover, both pathogenic conditions induce oxidative stress [27, 36], which oxidizes and unfolds proteins, after which they are easily ubiquitinated and pushed into the UPS pathway.

### 4.2. Insufficient Degradation of Proteins and Tissue Dysfunction

In contrast, modification of the 20S proteasome subunits by the lipid peroxidation product 4-hydroxy-2-nonenal (HNE), which occurs in cardiac ischemia/reperfusion, results in the selective inactivation of 20S activity [49]. Thus, modified and ubiquitinated proteins may accumulate to induce cell death in some pathological conditions. The UPS degrades numerous proteins, including apoptotic proteins, and regulates multiple signaling pathways. In human-dilated cardiomyopathy, the increased expression of the proapoptosis regulator p53 has recently been associated with UPS dysregulation and accumulation of polyubiquitinated proteins [50]. 

### 4.3. Dysregulation of UPS and Tissue Dysfunction

Therefore, the above findings show that the UPS, a selective and bulk protein degradation system, may be modified through multiple pathways. This system excessively degrades proteins necessary for tissue-specific function and/or cell survival, causing tissue pathogenesis. However, if this system is overwhelmed and/or dysregulated, modified proteins can accumulate and damage the cells. In inflammation, post-transcriptional molecular regulation involves several pathways that induce tissue dysfunction.

## 5. Potential Treatments of Diseases through UPS Regulation

Protein degradation damages tissue function, while it may also protect tissue from pathogenic protein accumulations. Since the UPS acts on specific proteins, regulating it may improve the prognosis. Bortezomib, a dipeptide boronic acid, is the first FDA-approved proteasome inhibitor for the treatment of multiple myeloma [51]. Bortezomib directly induces cell-cycle arrest and apoptosis, and it targets the tumor microenvironment. Combination chemotherapy regimens using Bortezomib have been developed that provide high rates of long-lasting remissions. Interestingly, in patients treated with Bortezomib, proteasome inhibition improves myocardial ischemia/reperfusion injuries, prevents postischemic ventricular tachyarrhythmias, promotes cardiac hypertrophy regression, and reverses diabetes-induced vascular endothelial dysfunction [52, 53]. Proteasome inhibition can be also applied locally. In a balloon injury model of the rat carotid artery, a locally administered proteasome inhibitor, MG132 [54] or lactacystin [55], significantly reduces atherosclerotic changes. In addition to Bortezomib, another proteasome inhibitor, Sorafenib has been also approved by FDA for advanced cancer therapy, and another candidate reagent is now under trial. In view of the potential therapeutic benefit of UPS regulation, its application to retinal diseases deserves further study.

## 6. Conclusions

Inflammatory retinal diseases, including diabetic retinopathy, induce inflammatory cytokines that influence protein metabolism. UPS-mediated protein degradation is a significant source of tissue dysfunction. Excessive degradation of tissue function essential proteins is an important factor in retinal neuronal dysfunction. Further analyses of the mechanisms that impair visual function may lead us to new therapeutic approaches for retinal neuroprotection.
